# Associations between visual-motor integration, pencil grip patterns, and holistic academic performance among school children

**DOI:** 10.1371/journal.pone.0351641

**Published:** 2026-06-11

**Authors:** Naufal Nordin, Woi Pui Juan, Nurul Umira Samsudin

**Affiliations:** Optometry and Vision Science Program, Center for Community Health Studies (ReaCH), Universiti Kebangsaan Malaysia, Kuala Lumpur, Malaysia; TIU: Tishk International University, IRAQ

## Abstract

**Background:**

Visual-motor integration (VMI) is a key developmental skill that supports children’s daily classroom tasks and academic readiness. This study examined the associations between VMI components, pencil grip patterns, and holistic academic performance among Malaysian primary school children.

**Materials and Methods:**

A total of 148 children (mean age = 9.94 ± 1.78 years) from three urban schools in Kuala Lumpur were assessed. VMI, visual perception, and motor coordination were measured using the Beery-Buktenica Developmental Test of Visual-Motor Integration (Beery-VMI). Pencil grip patterns were classified into five types using standardized photographs taken during the Beery-VMI assessment. Academic mastery in Malay Language, English, Science, and Mathematics was obtained from the Classroom-Based Assessment (CBA) system, which reflects holistic and continuous teacher-based evaluations.

**Results:**

Beery-VMI raw scores were significantly associated with CBA academic performance across all four core subjects, with the strongest correlations observed for Science (ρ = 0.332, p < 0.001) and Mathematics (ρ = 0.284, p < 0.001). Beery-VMI standard scores remained significantly associated only with Science (ρ = 0.305, p < 0.001) and Mathematics (ρ = 0.219, p = 0.008). This pattern suggests that raw scores reflect both VMI performance and age-related developmental factors relevant to academic performance, whereas standard scores provide age-adjusted estimates. Age significantly predicted VMI standard scores, accounting for 14% of the variance (R² = 0.140), with older children in this sample showing lower age-adjusted scores. Pencil grip differences were significant for VMI raw scores (χ² = 9.74, p = 0.045), with the lateral quadrupod grip demonstrating the highest performance.

**Conclusion:**

VMI was associated with academic mastery, especially in Science and Mathematics. Raw scores may reflect shared development, while standardized scores suggest a more specific age-independent link between VMI and academic performance. Pencil grip patterns showed limited association with VMI outcomes and may not strongly indicate visual-motor performance.

## Introduction

Visual-motor integration (VMI) is a fundamental developmental skill that enables children to coordinate visual information with fine-motor responses [1]. This skill forms the basis for many early academic and classroom tasks. Strong VMI skills support drawing, handwriting, copying, spatial alignment, and the manipulation of symbols that appear across the curriculum from the first years of schooling [2–4]. Research consistently shows that children with stronger VMI skills tend to perform better academically, particularly in subjects requiring spatial reasoning, symbol manipulation, and problem-solving such as Mathematics and Science [5–7]. VMI is often considered alongside related but distinct perceptual-motor abilities, including visual perception (VP) and motor coordination (MC). VP may support academic tasks that require children to recognize, discriminate, and interpret visual information, whereas MC may contribute to tasks that require accurate and controlled fine-motor output. Therefore, examining VMI together with VP and MC may provide a more comprehensive understanding of how perceptual and motor abilities relate to classroom learning. Despite this growing evidence, the relationship between VMI and holistic academic outcomes within non-exam-oriented systems remains underexplored.

Handwriting-related factors, including pencil grip, have also been widely discussed in relation to children’s VMI performance. Pencil grip is believed to influence mechanical efficiency, handwriting legibility, and motor control [8,9]. From a functional perspective, pencil grip may affect how efficiently a child translates visual information into written or drawn motor responses during classroom tasks. An inefficient or unstable grip may increase motor effort, reduce writing fluency, or affect the accuracy of fine-motor output, which may in turn influence performance on VMI-related tasks and written classroom work. Although the traditional tripod grasp is commonly emphasized, force-based evidence suggests that handwriting quality depends more on the child’s ability to maintain stable, coordinated digit forces than on the specific grasp pattern itself [10]. Thus, pencil grip may not directly determine academic achievement, but it may reflect or influence the motor-control demands involved in handwriting, copying, drawing, and other written academic activities. However, the extent to which different pencil grips relate to broader VMI outcomes and whether these relationships persist after adjusting for age has not yet been investigated. Clarifying this relationship is essential for guiding school-based screening and intervention practices, where grip correction is still commonly emphasized.

In Malaysia, academic performance is now evaluated through the Classroom-Based Assessment (CBA) system, which emphasizes cognitive, affective, and psychomotor development using mastery levels rather than traditional examination scores [11]. Although classroom assessment has been part of School-Based Assessment since 2011, it was restructured and officially renamed as Classroom Assessment in 2016. Its current implementation framework for Level 1 primary school pupils was strengthened with a new mandate beginning in 2019, reflecting a more holistic and continuous approach to monitoring student learning. However, no studies have examined how VMI skills, pencil grip patterns, and academic mastery interact within this context. Given these gaps, the present study investigates: (1) the relationship between VMI and supplemental test scores with academic mastery across core subjects, (2) the variation in VMI performance across different pencil grip patterns, and (3) the influence of age on VMI outcomes within this population. It was hypothesized that VMI would show the strongest positive association with academic mastery across core subjects, while VP and MC would have more domain-specific links. VP was expected to relate to visually demanding subjects, and MC to written or fine-motor tasks. VMI was also expected to vary by pencil grip pattern due to motor-efficiency demands, and age was expected to significantly influence VMI outcomes. Understanding these associations may provide information for early identification of children who may benefit from intervention and may inform school-based practices within the CBA framework.

## Materials & methods

### Study design and participants

This cross-sectional study involved 148 school children (N = 148; mean age = 9.94 years, SD = 1.78) from schools in Kuala Lumpur. This study employed a non-probability convenience sampling method at the school level. Three public urban primary schools in Kuala Lumpur were recruited based on accessibility and institutional approval. Eligible children attending these schools on the scheduled data collection days were invited to participate upon receipt of parental consent and child assent. Participant recruitment and data collection did not occur simultaneously across the three study sites. Each participating school was screened during one scheduled visit. However, the visits were conducted on different dates according to the school schedule. Recruitment at the first school was conducted on 6th June 2024, recruitment at the second school was conducted on 16th July 2024, and recruitment at the third school was conducted on 28th October 2024. The sample comprised 71 boys (48%) and 77 girls (52%). The children were enrolled in Year 1 through Year 6 and were drawn from multilevel classrooms. Children with developmental disorders, learning disabilities, or ocular/systemic conditions were excluded. Eligibility was determined based on teacher and parent/guardian report together with examiner observation during the assessment session. Formal diagnostic screening and medical record review were not part of the study protocol. To improve consistency, examiner observations followed the same assessment workflow for all children, and clarification was sought from teachers or parents/guardians when concerns about eligibility arose. All subjects were Malaysian and had functional understanding of both Malay and English, as these languages are taught in school. Data collection took place across the three participating schools between 6th June 2024 and 28th October 2024. Informed consent was obtained from parents or guardians, and assent was provided by the children prior to participation. The study was approved by the Universiti Kebangsaan Malaysia Research Ethics Committee (JEP-2024–515) and the Ministry of Education Malaysia (KPM.600-3/2/3-ERAS(20282). Authorization to conduct research in public schools within Kuala Lumpur was formally granted by the Federal Territory Kuala Lumpur State Education Department (Jabatan Pendidikan Wilayah Persekutuan Kuala Lumpur, JPN KL) through official written approval. Under this centralized approval system, the state education authority provides formal research authorization, while school administrators coordinate logistics and scheduling to ensure school activities are not disrupted. Documentation of the JPN KL approval has been submitted as a supporting file. Parents provided written informed consent and children gave assent before participation. They were informed that they could withdraw from the study at any time without penalty.

### Procedure

Before the main assessments, every child underwent a comprehensive vision screening to ensure adequate visual function. This included distance visual acuity at 6 meters, near visual acuity, and cover test. Distance visual acuity was measured using a Snellen chart, while near visual acuity was assessed using the standard near vision chart. Distance and near visual acuity were measured habitually. Children who did not achieve a visual acuity of at least 6/12 under habitual viewing conditions were excluded from the study.

Data collection was carried out during school hours on a scheduled date. All children were assessed in a single session, with most evaluations conducted individually and some in small groups of two to three children per examiner. Assessments were administered by final-year Bachelor of Optometry (B.Optom) students who were trained and supervised by registered optometrists with more than 5 years of experience. The evaluation included assessments of VMI skills, pencil grip patterns, and handedness. Holistic academic performance based on CBA was obtained from teachers once the most recent records became available.

### Measures

#### Visual-motor integration.

VMI skills were assessed using the Beery-Buktenica Developmental Test of Visual-Motor Integration, Sixth Edition (Beery-VMI) [1]. Because VMI was operationalized solely through this tool, the outcomes reported in this study refer specifically to performance on this standardized assessment rather than to broader visual-motor behaviors in classroom settings. The test consists of three subtests: Visual-Motor Integration (VMI), Visual Perception (VP), and Motor Coordination (MC). In the VMI subtest, children were asked to copy a sequence of geometric forms of increasing complexity. In the VP subtest, they identified matching forms among multiple choices without requiring a motor response. In the MC subtest, they traced shapes within narrow paths under a time constraint. The test yields raw scores, which can be converted into age-normed standard scores and percentile ranks. Both raw and standard scores were used in the present analysis. The Beery-VMI demonstrates strong psychometric properties, with test–retest reliability coefficients exceeding 0.85 and validity supported by correlations with academic achievement [1].

#### Pencil grip patterns and handedness.

Pencil grip patterns were assessed during the Beery-VMI tasks, as children were required to hold a pencil to complete the items. During the assessment, standardized photographs of each child’s pencil grip patterns were taken while the child was performing the task. To ensure consistency, photographs were taken from the same angle and distance for all subjects. The photographs were then reviewed by a trained examiner and used to classify pencil grip patterns into five predefined categories according to a previously described classification [12]: dynamic tripod, lateral tripod, dynamic quadrupod, lateral quadrupod, and others. Classification was conducted by a single trained examiner to maintain consistency. Handedness was recorded during the same task based on the hand used by the child to perform the Beery-VMI.

#### Holistic academic performance.

Holistic academic performance was obtained from the CBA system, an official framework used in all Malaysian schools to evaluate students’ progress in cognitive, affective, and psychomotor domains [11,13]. The CBA combines both formative assessment (ongoing teacher observations, projects, oral tasks, written work) and summative assessment. Teachers assign mastery levels using the mastery level scale, which consists of six levels: level 1 = minimal mastery, level 2 = basic mastery, level 3 = satisfactory mastery, level 4 = good mastery, level 5 = very good mastery, and level 6 = excellent mastery. Official school records provided by the teachers during the study were used as the source of CBA scores. CBA mastery levels are assigned by teachers using nationally defined performance standards and subject-specific descriptors issued by the Ministry of Education. As the curriculum, learning standards, and assessment framework are standardized nationally for public schools, the mastery levels are intended to be comparable across schools. No additional instructions were given to teachers for the purposes of this study; existing school CBA records were used. However, some variability in teacher interpretation and implementation may still occur. For this study, mastery level scores for four core subjects (Malay Language, English, Mathematics, and Science) were included in the analysis.

### Sample size estimation

Sample size was estimated using G*Power 3.1 for a two-tailed correlation test with an effect size of 0.30, α = 0.05, and power of 0.95. The required sample was 138, and the final sample of 148 was sufficient.

### Data analysis

All statistical analyses were performed using SPSS Version 29 (IBM Corp., Armonk, NY). Descriptive statistics were calculated to summarize the sample, including mean age, mean standard scores for VMI, VP, and MC, as well as frequencies for school subjects, handedness, gender, and pencil grip patterns.

Spearman’s rank-order correlations were conducted to examine associations between VMI and holistic academic achievement, with effect sizes reported using correlation coefficients (ρ). A simple linear regression analysis was used to determine whether age in months predicted VMI standard scores, and effect sizes were reported using R². Additionally, Kruskal-Wallis tests were used to compare Beery-VMI raw and standard scores (VMI, VP, and MC) across different pencil grip patterns. Statistical significance was set at p < 0.05.

## Results

A total of 148 subjects participated in the study, comprising 71 boys (48%) and 77 girls (52%), with a mean age of 9.94 years (SD = 1.78). Eleven subjects (7.4%) were left-handed, while 137 (92.6%) were right-handed. Pencil grip patterns showed that half of the subjects (50.0%) used a dynamic tripod grip, followed by lateral tripod (17.6%), dynamic quadrupod (13.5%), lateral quadrupod (12.2%), and others (6.8%). Academic performance based on CBA levels across four core subjects, together with demographic characteristics, handedness, and pencil grip distribution, are shown in Table 1.

**Table 1:** Demographic characteristics, handedness, pencil grip patterns, and academic performance.

Variable	n (%)
**Gender**	
Boys	71 (48.0)
Girls	77 (52.0)
**Handedness**	
Right-handed	137 (92.6)
Left-handed	11 (7.4)
**Pencil grip pattern**	
Dynamic tripod	74 (50.0)
Lateral tripod	26 (17.6)
Dynamic quadrupod	20 (13.5)
Lateral quadrupod	18 (12.2)
Other	10 (6.8)
**Academic performance (CBA)**	
**Malay Language**	
Level 1	0 (0)
Level 2	23 (15.5)
Level 3	47 (31.8)
Level 4	52 (35.1)
Level 5	26 (17.6)
Level 6	0 (0)
**English**	
Level 1	3 (2.0)
Level 2	7 (4.7)
Level 3	77 (52.0)
Level 4	39 (26.4)
Level 5	22 (14.9)
Level 6	0 (0)
**Science**	
Level 1	0 (0)
Level 2	14 (9.5)
Level 3	41 (27.7)
Level 4	48 (32.4)
Level 5	45 (30.4)
Level 6	0 (0)
**Mathematics**	
Level 1	0 (0)
Level 2	14 (9.5)
Level 3	46 (31.1)
Level 4	51 (34.5)
Level 5	37 (25.0)
Level 6	0 (0)

The mean, standard deviation, and median values for Beery-VMI raw and standard scores (VMI, VP, and MC), as well as age, are presented in Table 2. Both raw and standard scores are reported. An independent samples t-test showed no significant difference in VMI standard scores between boys (M = 86.06, SD = 12.50) and girls (M = 87.10, SD = 10.36), t(146) = –0.557, p = 0.578.

**Table 2:** Age and Beery-VMI scores.

Variable	Mean ± SD	Median
**Age (years)**	9.94 ± 1.78	–
**Beery-VMI (raw score)**		
VMI	19.11 ± 2.86	19.00
VP	21.10 ± 3.81	22.00
MC	22.92 ± 3.62	23.00
**Beery-VMI (standard score)**		
VMI	86.60 ± 11.41	87.00
VP	91.68 ± 17.17	95.00
MC	96.51 ± 14.57	97.00

A Kruskal-Wallis H test indicated significant differences in VMI standard scores across school grades, H(5) = 24.76, p < 0.001. Post-hoc comparisons with Bonferroni correction showed that children in Year 1 and 2 had significantly higher VMI scores compared to those in Year 4, 5, and 6.

Spearman’s correlation analysis revealed significant associations between Beery-VMI and academic performance (Table 3).

**Table 3:** Associations between VMI and academic performance across subjects.

Beery-VMI score	Academic Subject	Raw Scores ρ (p)	Standard Scores ρ (p)
**VMI**	Malay Language	0.278** (<0.001)	0.131 (0.112)
	English	0.261** (0.001)	0.116 (0.159)
	Science	0.332** (<0.001)	0.305** (<0.001)
	Mathematics	0.284** (<0.001)	0.219** (0.008)
**VP**	Malay Language	0.152 (0.064)	0.011 (0.898)
	English	0.220** (0.007)	0.059 (0.478)
	Science	0.135 (0.101)	0.120 (0.145)
	Mathematics	0.111 (0.180)	0.027 (0.743)
**MC**	Malay Language	0.162* (0.050)	0.052 (0.533)
	English	0.178* (0.031)	0.043 (0.600)
	Science	0.140 (0.090)	0.143 (0.083)
	Mathematics	0.146 (0.077)	0.074 (0.373)

Using raw scores, VMI showed weak positive correlations with Malay Language (ρ = 0.278, p < 0.001), English (ρ = 0.261, p = 0.001), and Mathematics (ρ = 0.284, p < 0.001), and a moderate positive correlation with Science (ρ = 0.332, p < 0.001). VP showed a weak positive correlation with English (ρ = 0.220, p = 0.007), while MC showed weak positive correlations with Malay Language (ρ = 0.162, p = 0.050) and English (ρ = 0.178, p = 0.031). Using standard scores, VMI remained moderately correlated with Science (ρ = 0.305, p < 0.001) and weakly correlated with Mathematics (ρ = 0.219, p = 0.008). Neither VP nor MC standard scores were associated with academic performance.

**Fig 1:**
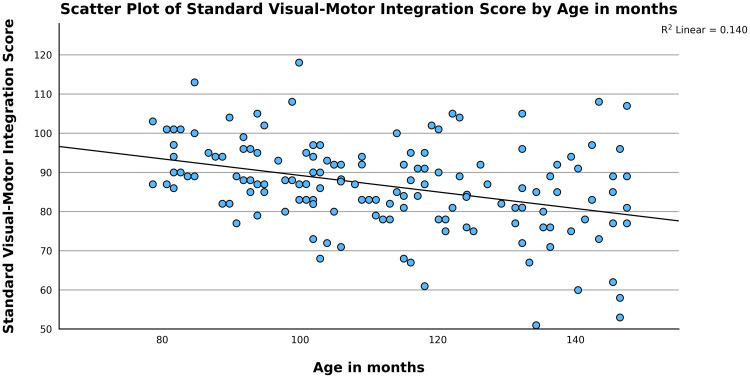
Relationship between age in months and VMI standard scores.

A simple linear regression showed that age in months significantly predicted VMI standard scores, F(1,146) = 23.78, p < 0.001, accounting for 14.0% of the variance (R² = 0.140). The regression equation was VMI = 110.84–0.22 × (Age in months), indicating that older children had lower VMI scores (B = −0.216, 95% CI [−0.303, −0.128], SE = 0.044, β = –0.374, p < 0.001). Fig 1 shows the scatter plot of subject’s VMI standard scores by age in months.

Kruskal-Wallis analysis of pencil grip patterns showed significant differences for VMI raw scores, χ²(4, N = 148) = 9.74, p = 0.045. Post hoc Bonferroni-adjusted pairwise comparisons revealed that the lateral quadrupod group scored significantly higher than the dynamic tripod group on VMI raw scores (adjusted p = .044). No other pairwise group differences were statistically significant. This effect was not significant for VMI standard scores, χ²(4, N = 148) = 5.66, p = 0.226. No significant group differences were observed for VP (raw: χ² = 7.30, p = 0.121; standard: χ² = 6.58, p = 0.160) or MC (raw: χ² = 6.54, p = 0.162; standard: χ² = 5.37, p = 0.251) (Table 4).

**Table 4:** Group differences in VMI (raw and standard scores) by pencil grip.

Beery-VMI score	χ² (Raw)	p (Raw)	χ² (Standard)	p (Standard)
**VMI**	9.74	0.045*	5.66	0.226
**VP**	7.30	0.121	6.58	0.160
**MC**	6.54	0.162	5.37	0.251

## Discussion

The present study discusses the importance of VMI in relation to children’s holistic academic performance. Beery-VMI scores were significantly associated with Science and Mathematics scores when assessed through the CBA system. Students with stronger VMI progress more smoothly in classroom tasks, while those with weaker skills may struggle despite adequate knowledge. More than a perceptual or motor skill, VMI integrates abilities that support learning readiness and participation in class [14,15]. In the context of Malaysia’s education system, these findings have important implications for the CBA framework. Designed to move beyond an exam-oriented system, the CBA captures holistic student development across cognitive, affective, and psychomotor domains [11]. This finding reinforces earlier reports that VMI skills are closely tied to academic achievement, particularly in subjects such as Science and Mathematics, which demand higher levels of visual processing and problem solving [5,16,17].

Associations between VMI and academic performance appeared more frequent when Beery-VMI raw scores were used than when standard scores were used. Raw scores correlated significantly with CBA levels across Malay Language, English, Science, and Mathematics, whereas standard-score associations persisted only for Science and Mathematics. This pattern is expected psychometrically because raw scores increase with age and therefore capture age-related developmental progression. Given the wide age range of the sample, correlations between raw VMI scores and academic performance may partly reflect shared developmental and schooling-related variance rather than a purely independent association between VMI and achievement. For this reason, the raw-score findings should not be interpreted as evidence that raw scores are superior to standard scores. Instead, they provide a descriptive indication of children’s functional task performance at the time of assessment. In contrast, standard scores adjust for age-based expectations and therefore offer a more conservative estimate of age-independent associations. The persistence of significant associations between VMI standard scores and Science and Mathematics suggests that VMI may be particularly relevant to subjects requiring visuospatial organization, symbol use, graphomotor coordination, and written layout. This may also explain why language subjects, unlike Science and Mathematics, appear more sensitive to learning exposure and classroom experiences than to age norms [18,19].

Our findings align with previous research [20], which reported significant associations between VMI and standardized test performance in English/ Language Arts and Mathematics. Evidence from another study [21] further showed that VMI components are domain-specific: visual perception and motor coordination predicted reading, all three components predicted Mathematics, and motor coordination predicted Written Language. Additionally, work in Grade 2 children [17] demonstrated that VMI remained the strongest predictor of both reading and Mathematics performance, even after accounting for visual acuity, stereoacuity, age, and socio-economic background. Collectively, these results show VMI’s contribution beyond motor skills and its role in supporting learning across subjects, consistent with our observation that VMI was linked to achievement in Science and Mathematics even within the CBA framework. While past studies analyzed standardized academic scores, our study extended this work by employing a CBA within a holistic framework that values growth across cognitive, affective, and psychomotor domains, thereby capturing dimensions of achievement not reflected in standardized measures.

Age was a significant negative predictor of VMI standard scores, with older children in this study obtaining lower scores relative to age-based norms, and the model accounting for 14% of the variance. This should not be interpreted as evidence that VMI skills declined with age in absolute terms. Instead, the findings suggest that within this sample of children, older children may not have performed as well as expected when compared with normative age-based standards. This pattern differs from typical developmental expectations, in which raw VMI ability generally improves with age [1,22,23]. Several contextual factors may hypothetically contribute to this pattern, including differences in access to enriched learning environments [24], opportunities for fine-motor practice [25,26], and academic support [27,28]. However, because socioeconomic status, home learning environment, and fine-motor exposure were not directly measured in this study, these explanations remain speculative. Taken together, the finding may indicate that age-related gains in VMI did not keep pace with normative expectations in this sample, potentially reflecting contextual differences in developmental exposure rather than a true decline in VMI ability.

Pencil grip groups differed significantly in VMI raw scores, with children using a lateral quadrupod grip showing the highest performance. However, these differences did not persist when standardized scores were examined, and no significant effects were observed for either VP or MC in both raw and standardized measures. This suggests that while certain grip patterns may confer a short-term advantage in visual-motor execution, they do not consistently translate into age-adjusted or broader visual-motor outcomes. A previous study reported that handwriting performance is more strongly associated with force control and fine MC than with grip style per se [9]. Their findings showed that older children, regardless of grip pattern, developed more stable and efficient force regulation, which in turn supported handwriting accuracy. This aligns with prior evidence indicating that variations in pencil grip do not necessarily hinder performance as long as the grip provides adequate stability and control [10]. Moreover, atypical grips, although less biomechanically efficient, may still allow functional output when compensatory strategies are adopted.

In this study, several limitations should be considered. First, the study was restricted to three public schools in urban Kuala Lumpur, which may not reflect the diversity of children from rural areas or different socioeconomic backgrounds. Second, this study did not assess refractive status, binocular vision anomalies, or accommodative function; therefore, the possible influence of these visual factors on VMI performance could not be determined. Third, academic performance was measured using CBA levels assigned by teachers, which, while holistic and widely implemented in Malaysian schools, may be influenced by teacher judgment and classroom practices rather than standardized testing. Fourth, VMI was assessed exclusively using the Beery-VMI instrument. While a standardized and widely used measure, it may not fully represent the range of dynamic visual-motor behaviors required in natural classroom tasks, meaning generalizations should be made cautiously. Finally, pencil grip pattern classification was conducted by a single trained examiner using standardized photographs. Although this approach supported procedural consistency, the absence of multiple raters meant that inter-rater reliability could not be assessed. Despite these limitations, the findings offer several important implications for education and clinical practice. The significant associations between VMI and academic performance, particularly in Science and Mathematics, suggest that visual-motor assessments could serve as a valuable tool for early identification of children at risk of underachievement. Importantly, the absence of strong links between pencil grip patterns and standardized VMI outcomes indicates that rigid correction of grip patterns may be unnecessary.

## Conclusion

In conclusion, this study suggested that VMI was associated with children’s holistic academic performance, with the most consistent associations observed for Science and Mathematics. Pencil grip patterns showed limited association with standardized outcomes, suggesting that grip patterns alone may not be a strong indicator of VMI performance. The observed negative association between age and VMI standard scores should be interpreted cautiously, as it may reflect lower performance relative to age-based norms rather than an actual decline in visual-motor ability. Together, these findings support the view that VMI is one of several skills contributing to school readiness and learning, and they support the inclusion of VMI assessment as part of broader early educational screening. Future work should build on these findings through longitudinal and contextually diverse studies, ensuring that children with visual-motor challenges are identified early and provided with appropriate multidisciplinary support.
