# Correction: Associations between visual-motor integration, pencil grip patterns, and holistic academic performance among school children

**DOI:** 10.1371/journal.pone.0356204

**Published:** 2026-08-14

**Authors:** Naufal Nordin, Pui Juan Woi, Nurul Umira Samsudin

The Funding statement for this article is incorrect. The correct Funding statement is as follows: This work was supported by Universiti Kebangsaan Malaysia under grant number GGPM-2024-064. The funder had no role in study design, data collection and analysis, decision to publish, or preparation of the manuscript.

The second author’s name is written incorrectly. The correct name is: Pui Juan Woi. The correct citation is: Nordin N, Woi PJ, Samsudin NU (2026) Associations between visual-motor integration, pencil grip patterns, and holistic academic performance among school children. PLoS One 21(6): e0351641. https://doi.org/10.1371/journal.pone.0351641.
